# Supplementary material for: BEN-solo factors partition active chromatin to ensure proper gene activation in Drosophila
Source: Nat Commun. 2019 Dec 13;10:5700. doi: 10.1038/s41467-019-13558-8 (PMC6911014; doi:10.1038/s41467-019-13558-8)
Supplement: Supplementary file 2 — Supplementary Information [file 41467_2019_13558_MOESM2_ESM.pdf]

BEN-solo factors partition active chromatin to ensure proper gene activation in *Drosophila*
by Ueberschär *et al.*

Description of Supplementary Figures, Tables and data

Supplementary Figure 1: Information of the *ELBA* and *insv* mutants, ChIP-seq controls and five categories of ChIP-seq regions**. (**Relevant to main Figure 1.)

Supplementary Figure 2: Common and Differential occupancy of the ELBA factors and Insv. **(**Relevant to main Figure 1 and 2.)

Supplementary Figure 3: Elba1 and Elba3 maintain genomic binding sites without the ELBA complex. (Relevant to main Figure 2.)

Supplementary Figure 4: Comparison of the ChIP-seq and the ChIP-nexus peaks. (Relevant to main Figure 3.)

Supplementary Figure 5: Expression changes of co-regulated and single factor regulated target genes. **(**Relevant to main Figure 4.)

Supplementary Figure 6: Known insulator motifs in co-occupied and single factor occupied genomic regions. (Relevant to main Figure 5.)

Supplementary Figure 7: The Elba factors insulate adjacent transcription units. (Relevant to main Figure 6.)

Supplementary Figure 8: Additional transgenes tested in the insulator assay. (Relevant to main Figure 7.)

Supplementary Table 1: Number of peaks called for each ChIP-seq datasets

Supplementary Table 2: Survival analysis of genetic interactions between elba/insv and insulator mutants

Supplementary Table 3: A summary of all tested insulator construct transgenes

Supplementary data 1: Deep sequencing library statistics

Supplementary data 2: High confident peaks from overlapping ChIP-seq and ChIP-nexus peaks

Supplementary data 3: Orientation Index of ChIP-nexus peaks

Supplementary data 4: RNA-seq data with differential expression

Supplementary data 5: Fold change between adjacent gene pairs from PRO-seq data

Supplementary data 6: list of reagents and oligos used in this study

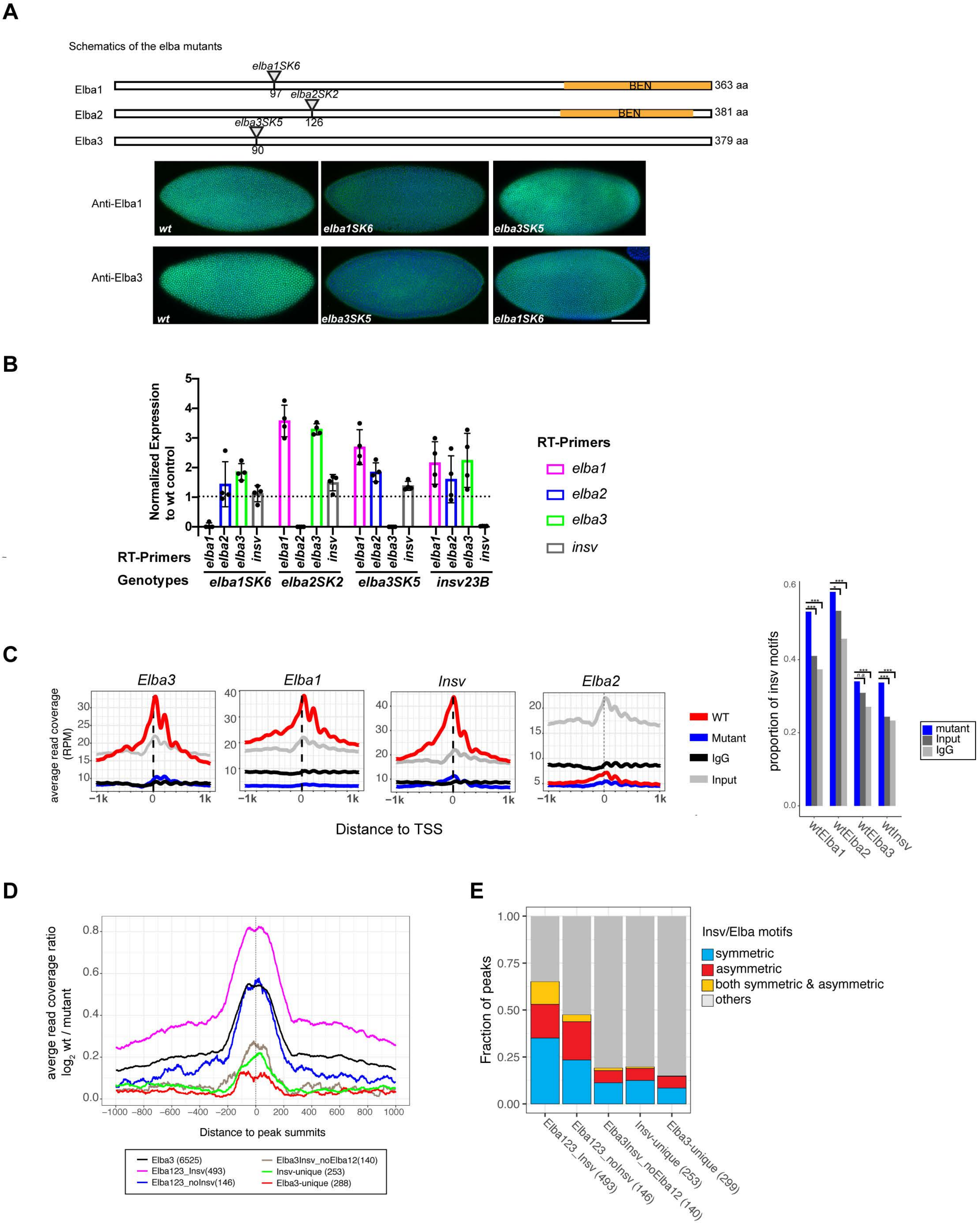
A
Schematics of the elba mutants
elba1SK6
Elba1
97
elba2SK2
BEN
363 aa
Elba2
126
BEN
381 aa
elba3SK5
Elba3
90
379 aa
Anti-Elba1
wt
elba1SK6
elba3SK5
Anti-Elba3
wt
elba3SK5
elba1SK6
B
Normalized Expression to wt control
RT-Primers
elba1 elba2 elba3 insv
Genotypes
elba1SK6
elba2SK2
elba3SK5
insv23B
RT-Primers
elba1
elba2
elba3
insv
C
Elba3
Elba1
Insv
Elba2
average read coverage (RPM)
Distance to TSS
WT
Mutant
IgG
Input
proportion of insv motifs
mutant
Input
IgG
wtElba1
wtElba2
wtElba3
wtInsv
D
averge read coverage ratio log_2 wt / mutant
Distance to peak summits
Elba3 (6525)
Elba123_Insv(493)
Elba123_noInsv(146)
Elba3Insv_noElba12(140)
Insv-unique (253)
Elba3-unique (288)
E
Fraction of peaks
Insv/Elba motifs
symmetric
asymmetric
both symmetric & asymmetric
others
Elba123_Insv (493)
Elba123_noInsv (146)
Elba3Insv_noElba12 (140)
Insv-unique (253)
Elba3-unique (299)

Supplementary Figure 1: Information of the *ELBA* mutants, ChIP-seq controls and five categories of ChIP-seq regions.

(A) Schematics of the disruption sites of the *ELBA* frame-shift mutations, and images of immunofluorescent staining on *wt*, *elba1* and *elba3* mutant embryos against the Elba1 and Elba3 antibodies (in green). Nuclear staining of Elba1 is lost in *elba1* mutant but present in other genotypes. Similarly, nuclear staining of Elba3 is only lost in *elba3* mutant. DAPI is used to label the nuclei (in blue). Scale bar: 100µm. **(B)** RT-qPCR result showing expression changes of the *ELBA* and *insv* genes in mutant versus *wt*. The Y-axis is plotted with averaged normalized value to *RPL32* and to wild-type from four biological replicates, each in technical triplicates. Note: the primers at the mutation failed to detect the wild-type transcripts in their cognate mutants; the genes do not reduce expression in any of the non-cognate mutants. Error bars represent Standard Deviation (S.D.). **C)** Normalized average coverage of the ChIP-seq signal in wild-type against mutant, IgG or Input centered at TSS is shown for each of the four ELBA/Insv factors. The fraction of motifs in the peaks indicates that the *wt* ChIP against mutant ChIP gave the highest motif enrichment. Statistics is calculated using Fisher's exact test (two-sided). * $p < 0.05$, * $p < 0.005$, *** $p < 0.0005$. **(D)** Comparison of average *wt*/mutant coverage ratio among five subsets: Elba3-unique, the three Elba factors but not Insv, Elba3 and Insv but not Elba1 and Elba2, all four factors, and Insv-unique. The subset that has four factor binding gave the highest average *wt*/mutant coverage while Elba3-unique gave the lowest. All sets were centered at the Elba3 peak summits except for Insv-unique, which is centered at the Insv peak summits. **(E)** Insv/ELBA motif fraction of these five Elba3 binding subsets.

Ueberschär_Supplemental_Fig.2

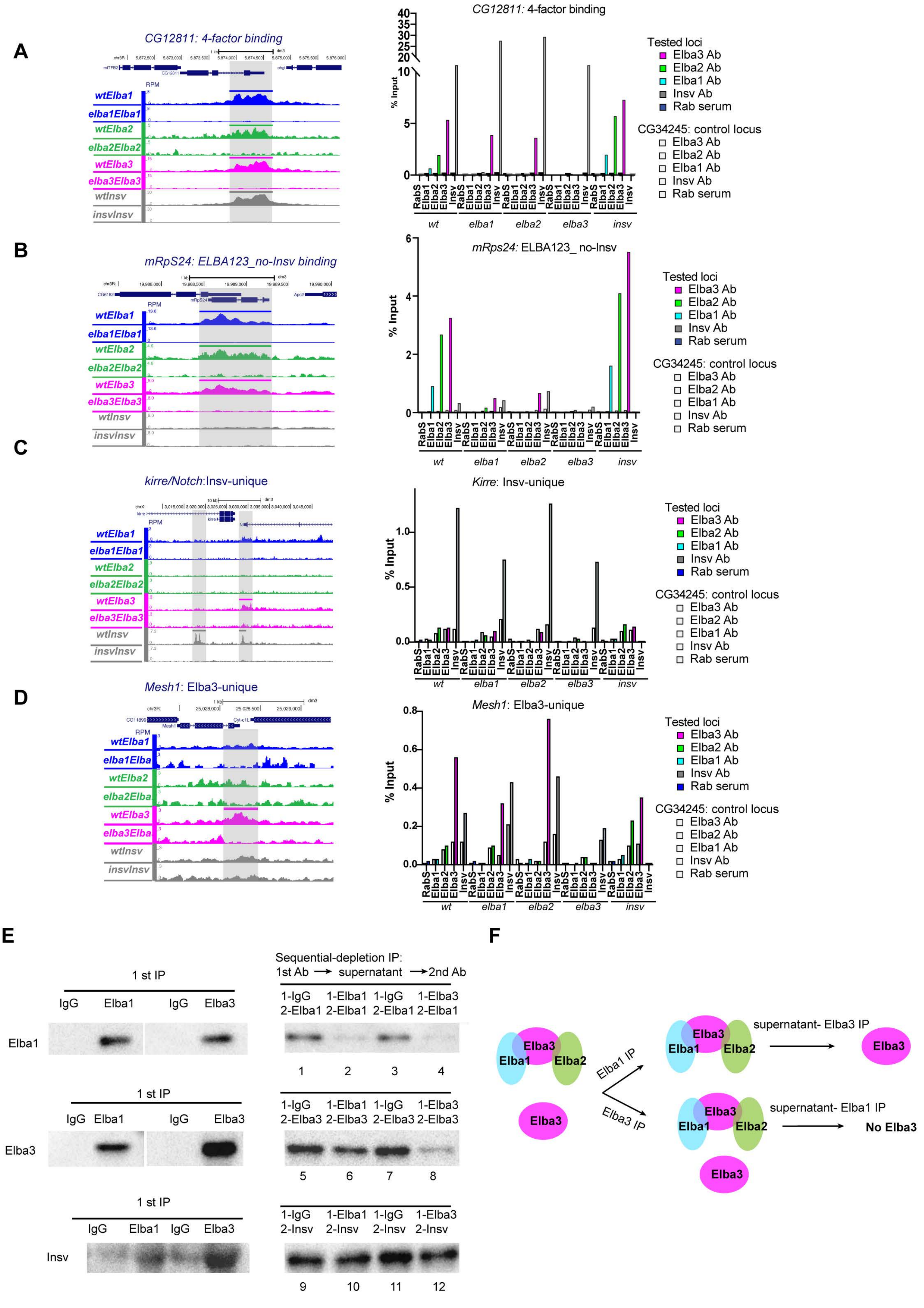

Supplementary Figure 2: Common and Differential occupancy of the ELBA factors and Insv.

(**A-D**) Four exemplary loci, including a 4-factor bound site in *CG12811* (A), an Elba1/2/3 without Insv co-binding site in *mRpS24* (B), an Insv-unique site in *Kirre/Notch* (C) and an Elba3-only site in *Mesh1*(D). The coverage tracks were normalized to the library sizes to give Read Per Million (RPM) per base. The ChIP-qPCR was performed using another set of antibodies for all the four factors and the results are plotted as %Input for each factor, averaging the technical triplicates. (**E**) Co-immunoprecipitation (co-IP) and sequential depletion immunoprecipitation (IP) experiments. IgG, the Elba1 and the Elba3 antibodies were used in the first IP, and the Elba1, the Elba3 and the Insv antibodies were used in the second IP. The blots from the first IP are shown on the left and those from the second IPs on the right. Note: small amount of Insv is also pulled down by Elba3. (**F**) A scheme to summarize the sequential depletion IP experiment. The Elba3 IP could deplete almost all the Elba1 molecules (lane 4), whereas the Elba1 IP could not deplete the Elba3 protein (lane 6).

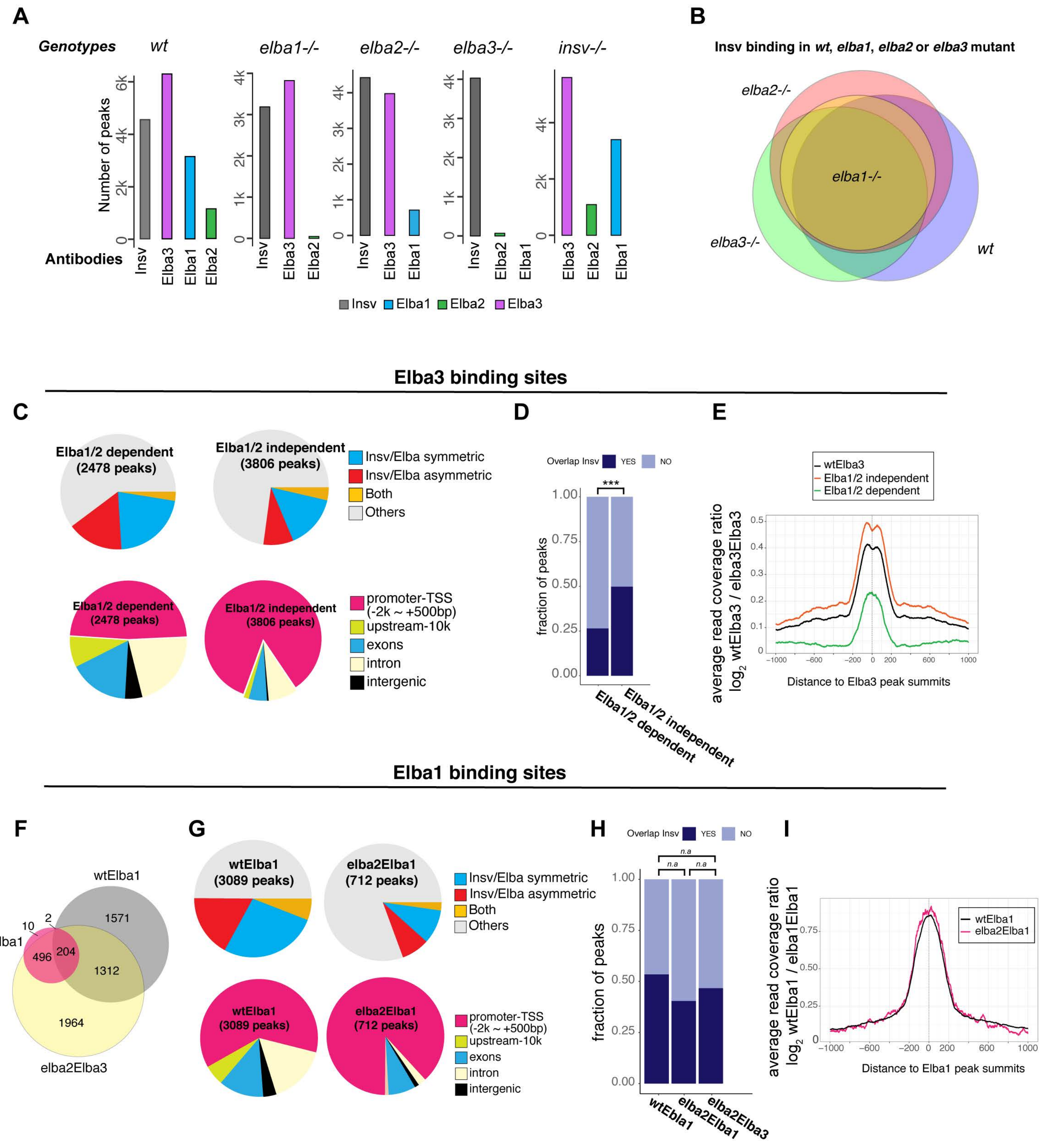
A
Genotypes
wt
elba1-/-
elba2-/-
elba3-/-
insv-/-
Number of peaks
Antibodies
Insv
Elba3
Elba1
Elba2
Insv Elba1 Elba2 Elba3
B
Insv binding in wt, elba1, elba2 or elba3 mutant
elba2-/-
elba1-/-
elba3-/-
wt
Elba3 binding sites
C
Elba1/2 dependent (2478 peaks)
Elba1/2 independent (3806 peaks)
Insv/Elba symmetric
Insv/Elba asymmetric
Both
Others
promoter-TSS (-2k ~ +500bp)
upstream-10k
exons
intron
intergenic
D
Overlap Insv YES NO
***
fraction of peaks
Elba1/2 dependent
Elba1/2 independent
E
wtElba3
Elba1/2 independent
Elba1/2 dependent
average read coverage ratio log2 wtElba3 / elba3Elba3
Distance to Elba3 peak summits
Elba1 binding sites
F
wtElba1
elba2Elba1
elba2Elba3
1571
1312
1964
496
204
10
2
G
wtElba1 (3089 peaks)
elba2Elba1 (712 peaks)
Insv/Elba symmetric
Insv/Elba asymmetric
Both
Others
promoter-TSS (-2k ~ +500bp)
upstream-10k
exons
intron
intergenic
H
Overlap Insv YES NO
n.a
fraction of peaks
wtEbla1
elba2Elba1
elba2Elba3
I
wtElba1
elba2Elba1
average read coverage ratio log2 wtElba1 / elba1Elba1
Distance to Elba1 peak summits

Supplementary Figure 3: Elba1 and Elba3 maintain genomic binding sites without the ELBA complex

(A) Re-grouping the number of peaks for each factor in Figure 2 to have the same genotype in each sub-figure. The number of peaks was obtained by using the ChIP-seq reads of *wt* against its own mutant ChIP. **(B)** Insv binding in three conditions: *wt*, *elba2*, and *elba3* mutants, showing that Insv binding does not rely on the ELBA factors. **(C)** The Elba1/2-dependent set of Elba3 binding sites has a higher fraction the the Insv/ELBA motifs compared to the Elba1/2-independent set. The Elba1/2-independent sites are enriched in promoter-proximal regions, while the Elba1/2-dependent sites in introns, exons, and distal regions. **(D)** 50% of the Elba1/2-independent Elba3 binding sites overlap with Insv sites while 25% Elba1/2-dependent ones overlap with Insv sites (P = 5.6E-78, Fisher's exact test, two-sided). **(E)** Average Elba3 *wt*/mutant coverage ratio of these two sets shows that the Elba1/2-independent set has significantly higher coverage than the Elba1/2-dependent set while the average read coverage for all Elba3 binding sites are in between. **(F)** Overlaps of Elba1 binding in *wt* and the *elba2* mutant as well as Elba3 in the *elba2* mutant. **(G)** Comparison of Elba1 binding in *wt* (wtElba1) and in the *elba2* mutant (elba2Elba1). elba2Elba1 has a smaller fraction containing the Insv/ELBA motif and is more enriched in the promoter-proximal regions compared to wtElba1. **(H)** Overlapping analysis of the Elba1 sites with the Insv sites shows that the elba2Elba1 set overlaps less with Insv than the wtElba1 set and the Elba3 sites in the *elba2* mutant (elba2Elba3), but did not reach statistical significance (P >0.05, Fisher's exact test, two-sided). **(I)** Comparison of average *wt*/mutant coverage ratio shows that the wt Elba1 sites, the Elba1 sites in the *elba2* mutant and the Elba3 sites in the *elba2* mutant have comparable coverage.

Ueberschär_Supplemental_Fig.4

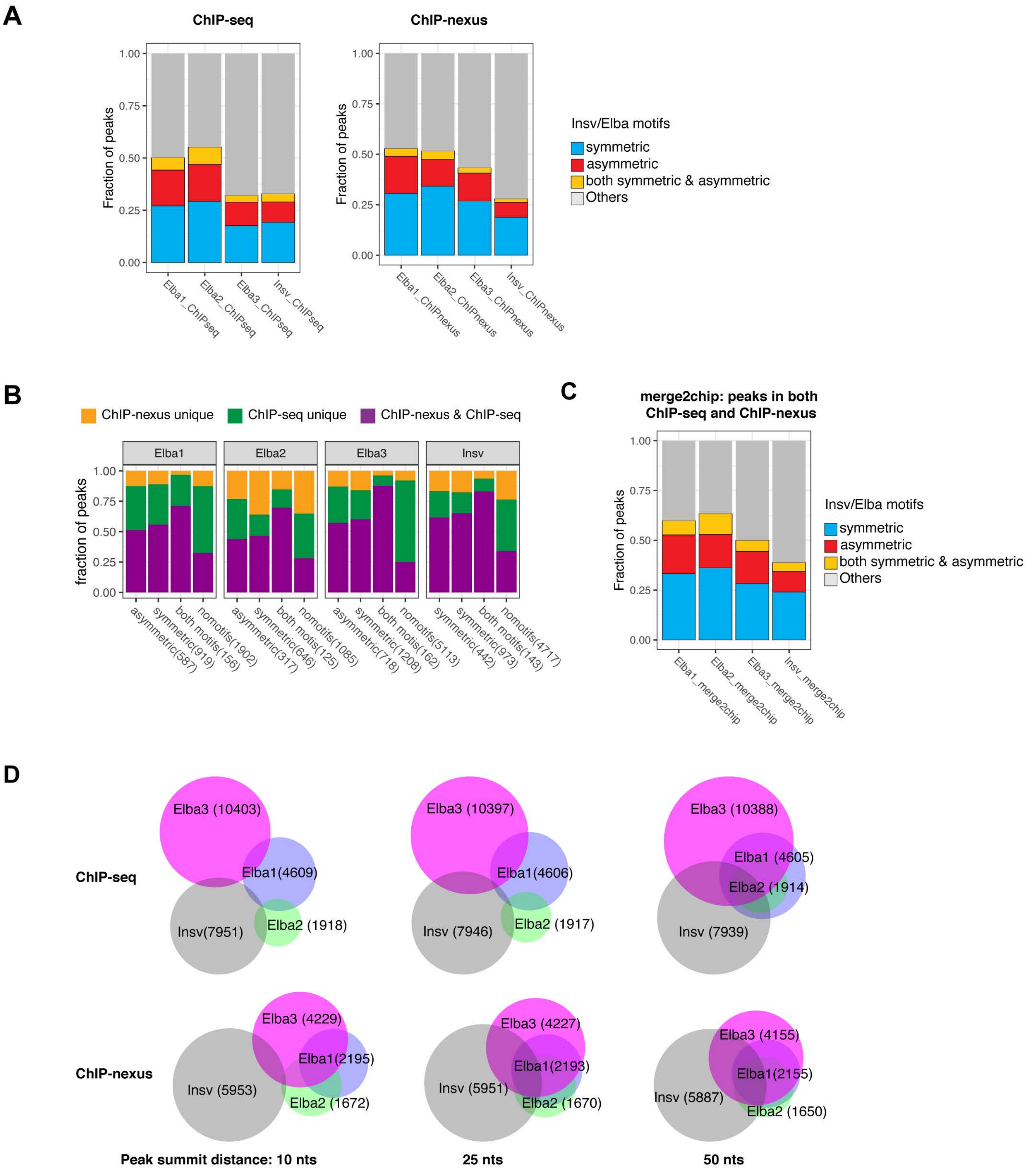

Supplementary Figure 4: Comparison of the ChIP-seq and the ChIP-nexus peaks.

(A) Motif enrichment frequency of the ChIP-nexus and the ChIP-seq data. A slight increase of motif enrichment frequency is observed for Elba3 with ChIP-nexus. **(B)** For each of the four factors, the motif occurrence frequency analysis for ChIP-seq and ChIP-nexus unique and overlapping peaks shows that the overlapping fraction has a higher frequency of motif occurrence (purple) than the ChIP-seq-unique (green) and ChIP-nexus unique (orange) sets. **(C)** Motif containing fractions in the ChIP-seq and ChIP-nexus overlapping sites are slightly higher than that in ChIP-seq and ChIP-nexus alone. **(D)** Peak overlapping analyses of the ChIP-seq and ChIP-nexus data within a given maximum distance (10nt, 25nt, or 50nt), by merging peak summits using "mergePeaks" function in Homer2 package. Note that as the peak calling program "MACS2" identifies multiple peak summits per peak, the number of peak summits illustrated here is larger than the number of peaks in Figure 1C.

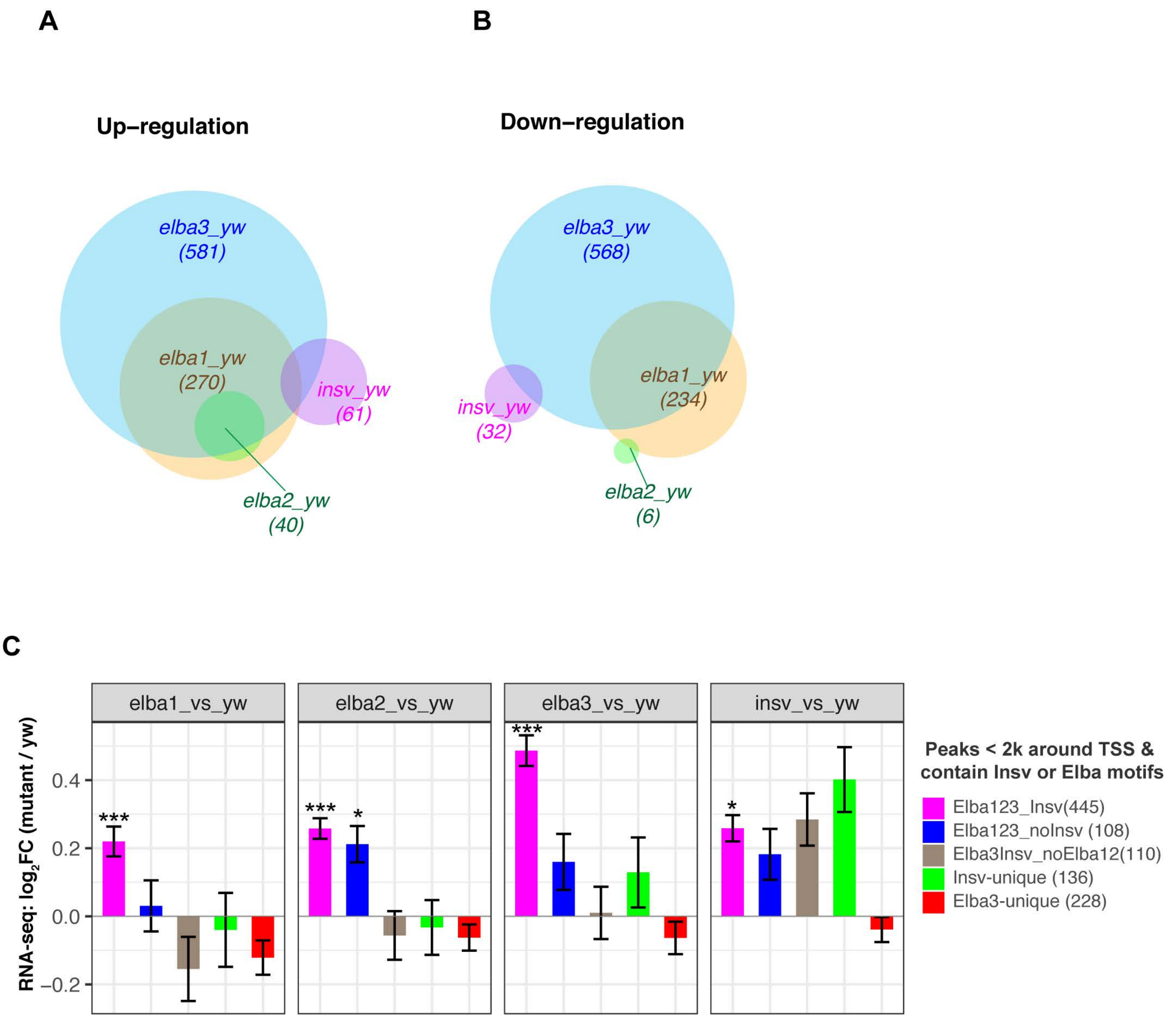

Supplementary Figure 5: Expression changes of co-regulated and single factor regulated target genes.

(A-B) For genes that contain ChIP-seq peaks at the promoter-proximal region (+/- 2KB), overlapping analyses are shown for differentially expressed genes in the four mutant genotypes versus wild-type (FDR< 0.2 and FC >1.5-fold): up-regulated **(A)** and down-regulated genes **(B)**. **(C)** Expression changes of the five binding subsets: genes bound by Elba3 alone (Elba3-unique), by the three ELBA factors without Insv (Elba123_noInsv), by Elba3 and Insv without Elba1 and Elba2 (Elba3Insv_noElba12), by all four factors (Elba123_Insv), and by Insv alone (Insv-unique). Gene set enrichment analysis (GSEA) was performed to test whether a peak set is statistically significantly up-regulated as a set (See Method). * p <0.05, ** p<0.01, *** p<0.001.

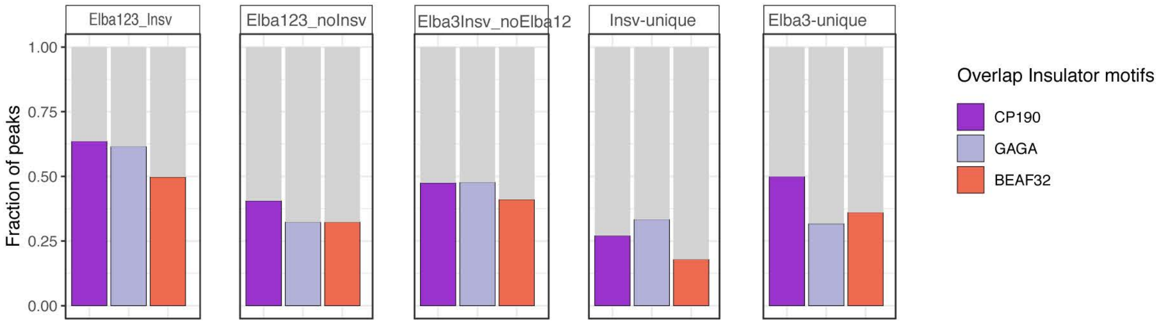

Supplementary Figure 6: Known insulator motifs in co-occupied and single factor occupied genomic regions.

Insulator motif enrichment in the peaks of the five binding subsets. The significantly enriched insulator motifs include binding sites for CP190, GAF and BEAF-32.

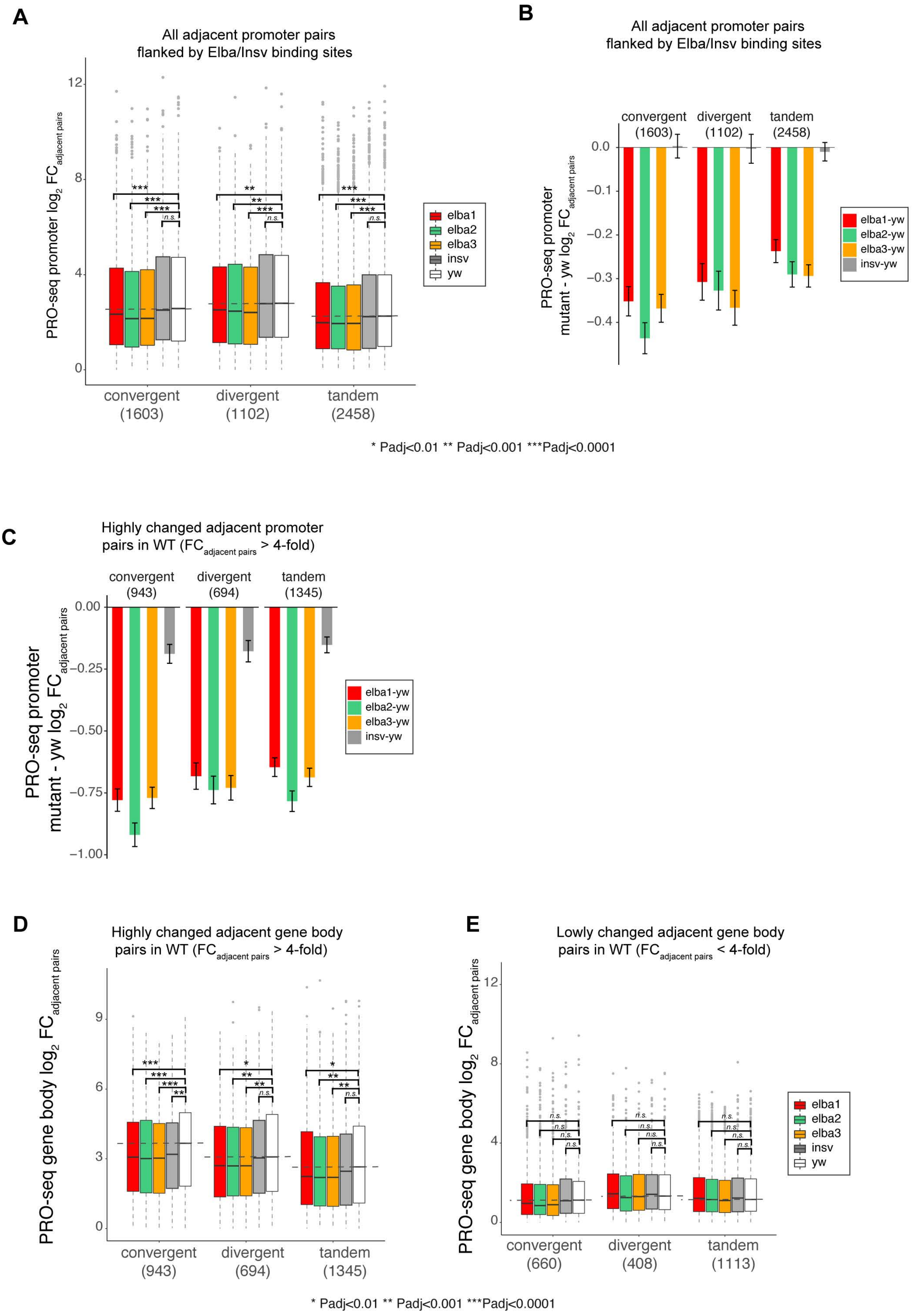
A
All adjacent promoter pairs
flanked by Elba/Insv binding sites
PRO-seq promoter log2 FCadjacent pairs
convergent (1603)
divergent (1102)
tandem (2458)
elba1
elba2
elba3
insv
yw
* Padj<0.01 ** Padj<0.001 ***Padj<0.0001
B
All adjacent promoter pairs
flanked by Elba/Insv binding sites
PRO-seq promoter
mutant - yw log2 FCadjacent pairs
elba1-yw
elba2-yw
elba3-yw
insv-yw
C
Highly changed adjacent promoter
pairs in WT (FCadjacent pairs > 4-fold)
convergent (943)
divergent (694)
tandem (1345)
D
Highly changed adjacent gene body
pairs in WT (FCadjacent pairs > 4-fold)
PRO-seq gene body log2 FCadjacent pairs
E
Lowly changed adjacent gene body
pairs in WT (FCadjacent pairs < 4-fold)
convergent (660)
divergent (408)
tandem (1113)
* Padj<0.01 ** Padj<0.001 ***Padj<0.0001

Supplementary Figure 7: The Elba factors insulate adjacent transcription units.

(A) A global reduction of PRO-seq expression change ($FC_{\text{adjacent pair}}$) between the adjacent promoters is detected in mutant compared to *wt* for the three ELBA factors but not for Insv. All three types of gene pair configuration, convergent, divergent, and tandem show a similar trend. **(B)** Quantification of the reduction in (A, all ELBA/Insv flanked promoters) shows the significant global reduction for the ELBA factors. **(C)** Quantification of the reduction for highly differentially expressed gene pairs (expression difference > 4-fold) in Figure 6A shows stronger effects than all the active pairs in (A). **(D-E)** Similar to the promoter pairs, using PRO-seq gene body expression, the expression difference between adjacent pairs is significantly reduced in the three *ELBA* mutants but not in *insv* mutant for the highly differentially expressed (> 4-fold) genes. No change is detected for the lowly differentially expressed (< 4-fold) genes. Statistical significance was calculated using two-tailed t-tests, and the p-values were adjusted by the Bonferroni multiple testing correction method (* $p < 0.01$, ** $p < 0.001$, *** $p < 0.0001$).

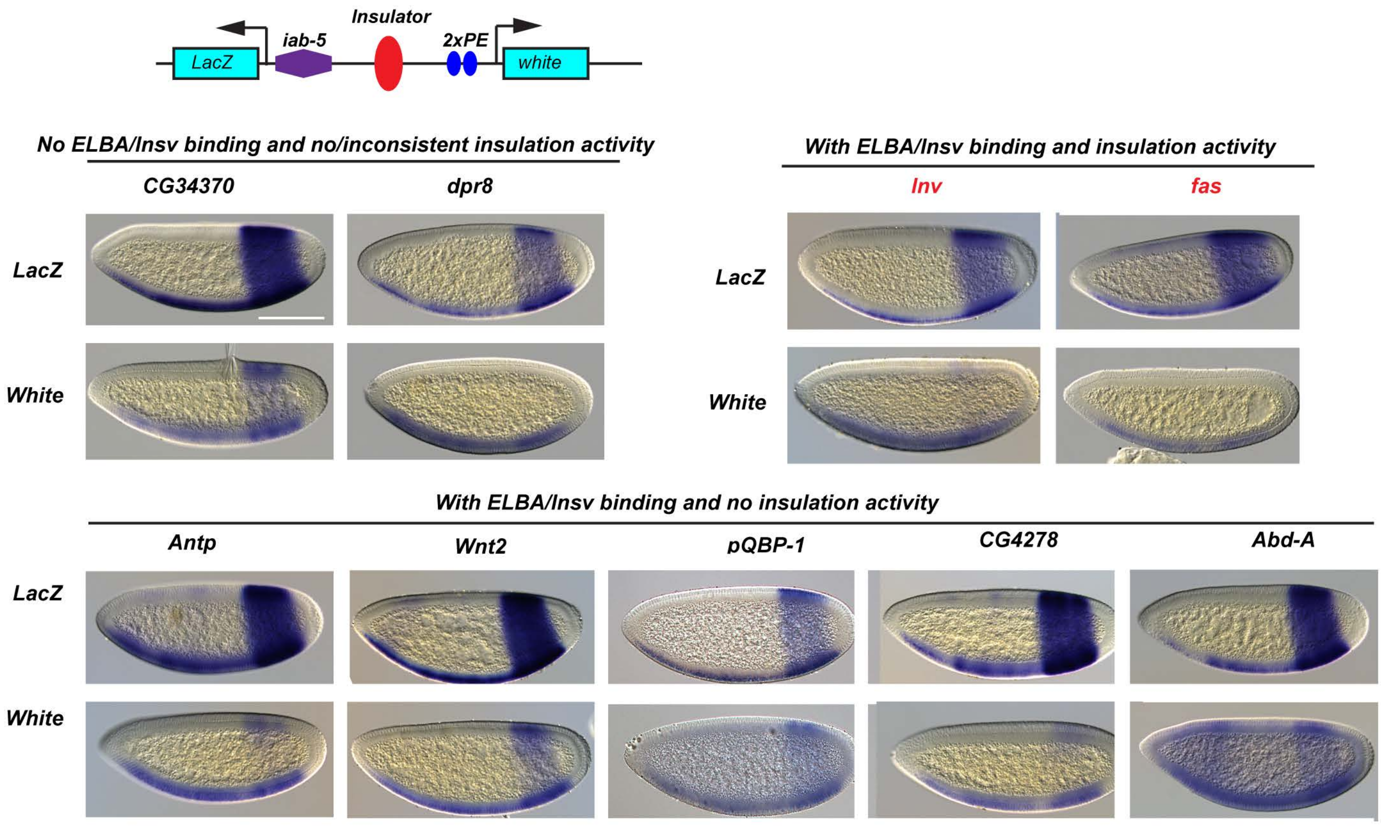

Supplementary Figure 8: Additional transgenes tested in the insulator assay.

The other transgenes in the transgenic insulator assay. *In situ* hybridization images show expression of the *lacZ* and the *white* genes is driven by the *2xPE* and the *iab-5* enhancers. The reporters with detectable effects are highlighted in red. Scale bar: 100 uM.

Supplementary Tables

Supplementary Table1: Number of peaks called for each ChIP-seq datasets

antibody	ChIP	control	npeaks
Elba1	wtElba1	elba1Elba1	3089
	elba2Elba1	elba1Elba1	712
	elba3Elba1	elba1Elba1	0
	insvElba1	elba1Elba1	3397
	wtElba1	wtIgG	8377
	wtElba1	wtInput	9077
Elba2	wtElba2	elba2Elba2	1454
	elba1Elba2	elba2Elba2	48
	elba3Elba2	elba2Elba2	68
	insvElba2	elba2Elba2	1092
	wtElba2	wtIgG	3027
	wtElba2	wtInput	1971
Elba3	wtElba3	elba3Elba3	6284
	elba1Elba3	elba3Elba3	3825
	elba2Elba3	elba3Elba3	3976
	insvElba3	elba3Elba3	5606
	wtElba3	wtIgG	9523
	wtElba3	wtInput	9746
Insv	wtInsv	insvInsv	4579
	elba1Insv	insvInsv	3188
	elba2Insv	insvInsv	4423
	elba3Insv	insvInsv	4030
	wtInsv	wtIgG	8626
	wtInsv	wtInput	8451

Supplementary Table 2: Survival analysis of genetic interactions between elba/insv and insulator mutants

Genotypes	Viability
**GAGA factor/*Trl***	
elba1/elba1;FRT Trl R85/TM3Sb	viable
elba2/elba2;FRT Trl R85/TM3Sb	lethal
elba2/elba2;FRT Trl R85/pBac(insvelba2)	viable
elba2/elba2;Trl 13C/TM3Sb	viable
elba3/elba3;FRT Trl R85/TM3Sb	lethal
elba3/elba3;Trl 13C/TM3Sb	viable
insv/insv;FRT Trl R85/TM3Sb	viable
CP190	
elba1/elba1;CP190P11/TM3Sb	lethal
elba2/elba2;CP190P11/TM3Sb	viable
elba3/elba3;CP190P11/TM3Sb	lethal
insv/insv;CP190P11/TM3Sb	viable

Supplementary Table 3: A summary of all tested insulator construct transgenes

Fragment	Insulation	multiple lines	Elba-bound	Insv-bound	Elba motif
**µMAR**	N	n			
**Abd-A**	N	n	+	+	-
**CG4278**	N	n	+	+	CCAATAAG
**Antp**	N	n	Elba3	+	-
**PQBP-1**	N	n	weak Elba3	+	-
**Wnt2**	N	n	+	+	-
**inv**	**Y**	y	+	+	-
**fas**	y	y	weak	+	-
**Lasp**	**Y directional**	y	Elba3, weak Elba1	+	-
**wg**	**Y**	y	++	+	CCAATAAG
**CG42368**	y	y	+	+	-
**Parp**	**Y**	y	+	++	CTTATTGGTCTTATTGG
**CG34370**	N	n	-	-	-
**CG32333**	N	n	-	-	-
**dpr8**	y	inconsistent	-	-	-
