# Supplementary material for: BEN-solo factors partition active chromatin to ensure proper gene activation in Drosophila
Source: Nat Commun. 2019 Dec 13;10:5700. doi: 10.1038/s41467-019-13558-8 (PMC6911014; doi:10.1038/s41467-019-13558-8)
Supplement: Supplementary file 3 — Description of Additional Supplementary Files [file 41467_2019_13558_MOESM3_ESM.pdf]

Description of Additional Supplementary Files

File Name: Supplementary Data 1
Description: Deep sequencing library statistics.

File Name: Supplementary Data 2
Description: High confident peaks from overlapping ChIP-seq and ChIP-nexus peaks.

File Name: Supplementary Data 3
Description: Orientation Index of ChIP-nexus peaks.

File Name: Supplementary Data 4
Description: RNA-seq data with differential expression.

File Name: Supplementary Data 5
Description: Fold change between adjacent gene pairs from PRO-seq data.

File Name: Supplementary Data 6
Description: List of reagents and oligos used in this study.
